# Investigating the role of internet-based educational application in the dental sciences

**DOI:** 10.1016/j.heliyon.2023.e23643

**Published:** 2023-12-13

**Authors:** Samar A. Alsaleh, Abeer S. Alzawawi, Alanood A. Alzuhair, Sara A. Kalagi, Ebtissam M. Al-Madi, Ashit Kumar Dutta

**Affiliations:** a Department of Prosthetic Dental Sciences, College of Dentistry, King Saud University, Riyadh, Saudi Arabia; b Department of Periodontics and Community Dentistry, College of Dentistry, King Saud University, Riyadh, Saudi Arabia; c Assistant Consultant in Advanced General Dentistry at Ministry of National Guard Health Affairs (MNG-HA), Riyadh, Saudi Arabia; d Lecturer in Restorative Dentistry, Restorative and Prosthetic Dental Sciences Department, College of Dentistry, King Saud Bin Abdulaziz University for Health Sciences, Riyadh, Saudi Arabia; e Department of Restorative Dental Sciences, College of Dentistry, King Saud University, Riyadh, Saudi Arabia; f Department of Computer Science and Information Systems, College of Applied Sciences, AlMaarefa University, Ad Diriyah, Riyadh, 13713, Kingdom of Saudi Arabia

## Abstract

The study intended to identify the significance of the online information retrieval system (IRS) in evidence-based dentistry (EBD). Thus, the researchers apply a set of pre-and post-tests to evaluate the current knowledge of clinicians and students on online IRS. **Materials and Methods**: The researchers followed repeated measure design in this study. They applied random sampling technique for conducting pre-and post-test assessment. Five scenarios based EBD were developed to evaluate the performance of the participants. The researchers employed two phases in order to achieve the study's objective. In the first phase, 98 clinicians and 70 students were invited to attend three out of five clinical scenarios using IRS. In the second phase, the participants were invited to participate in a 15-min lecture presented by the researchers on the searching strategies and guidelines to apply keywords for searching the evidence using IRS. A significant level of p < 0.05 was obtained from the statistical analysis using the SPSS program version 16. **Results:** Of the 98 clinicians, only 37 responded to the questionnaire, with a response rate of 37.8 %. On the other hand, out of 70 students, 23 responded to the questionnaire, with a response rate of 32.8 %. In the pre-test, clinicians and students correctly answered 58.3 % of scenario questions. However, the data analysis outcome revealed that only 40.5 % of participants provided a relevant evidence source after an internet search. The students spent an average of 9 min to complete the task, whereas clinicians spent 16 min. After the completion of the lecture, 23 students and clinicians responded to the pre-test, whereas 10 responded to the post-test. Most students believed that the lecture was helpful and recommended similar types of lectures to be presented in the curriculum. The study findings highlight that the percentage of evidence provided in the "pre-test" was 60 %, which was improved in the post-test to 73.3 %. **Conclusion**: The experimental outcome suggests that internet-based educational applications enhance students' learning strategies. Additionally, the IRS supports clinicians in retrieving effective materials for treating their patients. Furthemore, there is a demand for extracurricular activity to improve the search strategies of clinicians and students to strengthen EBD.

## 1 Introduction

The Internet era generated a revolutionary impact on students' learning practices [1]. Medical information systems cover a wide range of diseases, treatment procedures, self-care, and pharmaceutical product information [2]. However, the web conceals several flaws, including the variable quality of medical information and the challenges in locating and comprehending this information [3]. Online sources of medical knowledge abound, with content produced by people from many occupations and with a wide range of motivations and perspectives [[4], [5], [6], [7]]. It can be difficult for students and educators in medicine due to the overwhelming quantity and disorganized nature of the accessible material. They need to swiftly and accurately obtain relevant, up-to-date data. There is a demand for investigating the students' perception of using online libraries and other information sources. Students increasingly rely on the Internet as a reliable source of particular information for their schoolwork. Students may take a more customized for retrieving the useful information from the online IRS. Most students go online without any sort of guidance or instruction. The skills and knowledge needed to navigate the web differ greatly from those needed to use a print or conventional library. The IRS is not well structured, and retrieving targeted information from it is becoming increasingly difficult.

To be successful dentists and researchers, dental clinicians and students require an understanding of search strategies to retrieve dental information. IRS encompasses acquiring, organizing, and searching for knowledge-based information [9]. Research studies published in journals, papers, and other sources are the primary sources. Secondary sources of knowledge are scholarly works that analyze, summarize, or synthesize findings from the primary literature. Books, review articles, and lecture notes are the most typical forms of literature that fall under this category. Secondary sources are readily available in the form of guidelines for healthcare practices, web data, expert comments, and wallet manuals in dental specialities [10]. There is an argument that secondary literature is the most frequently used by practitioners in the medical and dental fields.

Knowledge-based information may serve various purposes. Finding particular facts or answers to issues may be accomplished rapidly with minimal effort by consulting secondary knowledge-based resources. As a result of the widespread availability of low-quality material online, several groups have created electronic standards and online tools specifically for healthcare professionals. Recent studies showed that information retrieval systems can improve clinicians' decision-making ability.

When a clinical concern occurs, online information systems can access the most recent available data. They can potentially be one of the most useful strategies for encouraging evidence-based medicine in clinical settings [11]. Over 50 % of dental clinical issues may be answered by searching databases like Pubmed, MEDLINE, etc [12]. Many clinicians take advantage of point-of-care online IRS for decision-making and patient care. The research methodology and search strategies have recently been introduced in dental postgraduate studies. Students rely on research portals to extract practical research ideas to extend their research in dental sciences. However, there is a lack of expertise for clinicians and students to extract the relevant materials to make effective decisions. Previous research has been hindered by its reliance on a student sample, information retrieval methods, which include a single resource, and an inability to mimic the time limitations in healthcare activities.

Commonly consulted resources (including colleagues, the Saudi digital library, personal lesson notes, and the Current care Recommendations) are readily available and typically comprise summarized guidelines [13]. Positive sentiments toward these resources among students are to be expected. There might be several reasons why students do not frequently seek out scholarly litearatue. Dental publications rely heavily on statistical methodologies [13]. In the past decades, there has been a rise in research publications using statistical approaches. Most pre-professional medical and dentistry schools fail to adequately prepare their students for the realities of clinical practice [14]. This confusion might discourage people from looking for answers in scholarly literature. This prompts the topic of whether or not authors should stress the findings in their publications clearly and succinctly. There may also be issues with getting your hands on the whole content. The exponential growth of open-access journals provides students with unlimited access to scholarly material to improve their knowledge.

Research shows that mobile phones and software are widely used in medical instruction. It has the potential to enhance both the educational process and the outcomes for patients. Most importantly, instructional software helps students save time and gain more value from their study sessions. Educators have turned to various digital tools to keep up with the ever-increasing volume of dental literature [14]. Several software programs for oral ulcers and systemic disorders have been developed. However, a few applications are related to the dental sciences. Medical technology has improved disease control and life expectancy, allowing more individuals with systemic disorders to maintain their lives. To safely treat these individuals, dentists need to adapt their modern approaches [15]. A lack of understanding in this area might have permanent implications. Dentists should be competent to diagnose and prescribe therapy. As healthcare professionals, they need to be ready to control medical emergencies and have sufficient information about drug interactions and side effects of drugs to treat patients promptly [15]. In addition, one of the challenges confronted by the educational system is the lack of adequate abilities in searching for relevant resources. Therefore, the students must strengthen their information retrieval skills to extract insights from the large volume of data.

Dentists require expertise to efficiently retrieve knowledge from a pool of literature [14]. It appears that undergraduate dental and medical programs in Saudi Arabia have advanced to teach students how to find and use relevant research materials. Saudi Arabia has made great strides in implementing information technology into its medical and dentistry bibliographical databases [15]. However, students and clinicians require additional time to retrieve the relevant database information. The dentistry curriculum motivates students to read full-text publications to bolster their utilization of primary knowledge-based material [16]. The retrieval of information plays a crucial role in supporting evidence-based practice. Clinicians are required to make well-informed judgments on patient care by relying on the most reliable data derived from research studies and clinical guidelines. The ability to effectively obtain, assess, and utilize pertinent medical literature is crucial in order to provide effective healthcare service [17]. In addition, as dental school draws to a close, the clinical component should include additional instruction and a variety of knowledge retrieval tasks. Students' usage of libraries and other information sources has been shown to rise when they are given proper instruction in extracting information [19]. These abilities are crucial in the critical appraisal process, which involves analyzing research to determine its reliability and applicability in a dental setting.

In the field of dentistry, the ability to retrieve information is critical for being up-to-date, making choices based on evidence, teaching patients, carrying out research, and delivering high-quality dental treatment. It assists dental professionals at all phases of their employment, from classroom instruction through in-office procedures and laboratory investigations.

The study's objectives are.

1. To identify the significance of the IRS in dental science,
2. To assess the current IRS knowledge of Saudi dentists and dental students,
3. To evaluate the validity and integrity of the participant's responses.

## 2 Research methodology

According to the study's objective, the authors develop a hypothesis stating that online information retrieval systems support dental clinicians and students to improve their research and decision-making skills. In order to evaluate the validity of the hypothesis, we experimented with experienced clinicians who are now in active practice and postgraduate students. The experiment aimed to determine whether utilizing an online information retrieval system increases a clinician's and student's performance when providing answers to clinical questions within a certain amount of time.

Pre-tests and post-tests are widely recognized as excellent assessment tools in the field of education. These assessments serve several purposes, including measuring student learning, providing significant insights for instructional decision-making, and analyzing the overall effectiveness of teaching and learning activities. In social studies, educators play a crucial role in facilitating students' acquisition of a more profound comprehension of historical occurrences, societal frameworks, and other significant subject matters.The pre-post test design entails the selection of a representative sample from a specified population, followed by evaluating participants' conditions before and after an intervention or therapy. This methodology enables researchers to assess the effects of the intervention on a consistent cohort of individuals throughout a longitudinal period. The use of identical participants for both the pre-test and post-test is crucial in this design due to its ability to facilitate the evaluation of individual changes over a period of time. The intervention's efficacy is evaluated by comparing baseline data with post-intervention assessments of the same subjects.

### 2.1 Participants

The target population of the study is the clinicians and dental science students. We obtained ethics approval (IRB23-019) from AlMaarefa University, Riyadh, Saudi Arabia for conducting this research. The participants consisted of clinicians and students. The clinicians (Interns, Demonstrators, Postgraduates [AEGD, Board, and Specialists]) practice in different hospitals, academic institutions, and private clinics in Riyadh province, Saudi Arabia. In addition, students studying at different levels at King Saud University. (third year, fourth year, and fifth year) were included. These clinicians and students were contacted over two months and were given consent and test forms.

### 2.2 Procedure

**Fig. 1:**
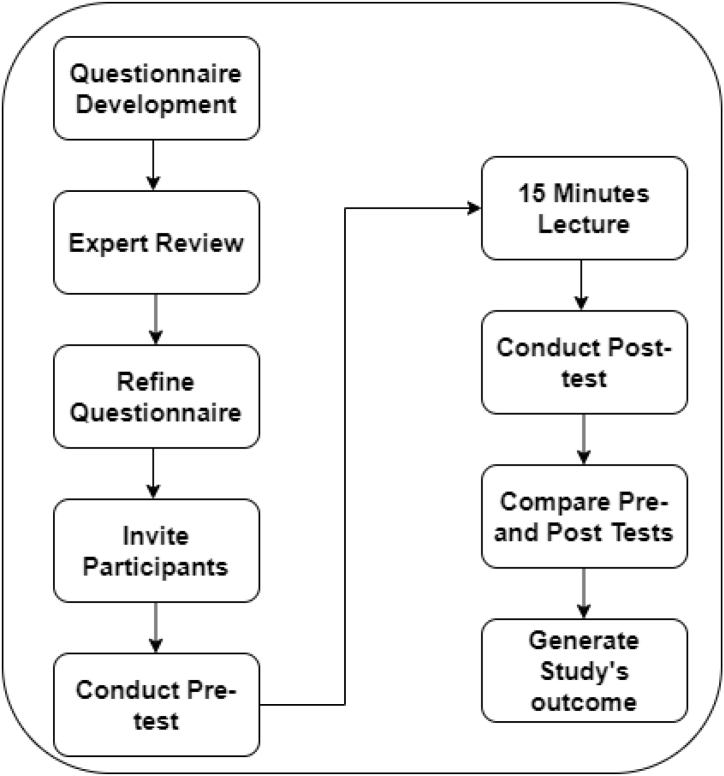
Research methodology.

In this study, the researchers followed repeated measure design (Pre-and post-test measure design). A random sampling technique was applied to invite the participants to pre- and post-test assessments. Based on the hypothesis, the authors developed the questionnaire to identify the clinician's and student's current knowledge of the IRS based on evidence based dentistry. Fig. 1 presents the proposed research methodology. The demographic data includes gender, specialization, affiliation, and postgraduate course level) and professional (years spent practicing prosthodontics/restorative and weekly hours). The researchers In addition, the three clinical decision-making approaches, patient-related and clinical case-related, are included in the questionnaire. A set of scenarios is developed to test the clinicians' and students' expertise in information retrieval skills. Frequencies and percentages were used to describe the participants' characteristics, while means, standard deviations, and medians were used to describe quantitative variables with a normal distribution.

Furthermore, the authors employ a coding system to identify the significant improvement of the participants in pre- and post-tests. The study consisted of two parts: In part 1, clinicians and students were asked to participate in the study to investigate their use of online information retrieval systems. Initially, written consent was obtained from the participants. Furthermore, a form containing demographic data (age, gender, academic year, place of work, specialty, year of graduation, college of which graduated, and academic year). In addition, the current level of computer skills is rated on a five-point scale (poor, fair, good, very good, excellent). Finally, the frequency of employing online information retrieval systems using a five-point scale (never, once per month, two to three times per month, once per week, two to six times per week). Table 1 shows the inclusion and exclusion criteria of the proposed study.

**Table 1:** Inclusion and exclusion criteria.

Inclusion Criteria	Exclusion Criteria
Clinicians who have experience of more than one year in EBD.	Non dental science clinicians and administrators of dental care centers.
Students who have experience in IRS and computer skills.	First and second year dental science students.

#### 2.2.1 Scenarios

Evidence-based dentistry is a method where dentists use the most recent and reliable scientific information to guide their clinical judgments [13]. The process entails the amalgamation of clinical competence, contemporary research results, and patient preferences in order to administer dental treatment that is optimum in nature. For instance, the dental professional is confronted with a patient exhibiting a molar tooth with a profound cavity. The individual is encountering pain and discomfort and actively seeking medical intervention. The dental professional starts the evaluation process by examining the patient's dental status, encompassing the magnitude of the dental caries, the manifestation of any symptoms, and the general state of oral well-being.

The dentist thoroughly examines the available literature and dental recommendations for managing deep caries and dental caries. The individuals involved in the consultation process rely on credible sources, including peer-reviewed research publications and clinical practice guidelines endorsed by dentistry societies.Drawing upon the literature research findings and the clinical examination results, the dentist discerns many treatment alternatives for the patient. These potential alternatives may encompass:

• The dental filling, also known as a restoration, is utilized to fix the structure of a tooth.
• Root canal treatment is used when dental caries have progressed to involve the tooth pulp.
• The tooth extraction is necessary when it is determined that the tooth cannot be preserved.

The dental professional engages in a comprehensive discussion with the patient on the available treatment alternatives, elucidating the potential advantages, hazards, and results associated with each option. The dentist considers the patient's choices, medical history, and concerns.Following an extensive dialogue between the dental practitioner and the patient, a collaborative decision is reached on the optimal course of treatment. The most reliable and up-to-date evidence influences this decision, the specific requirements of the patient, and the provision of informed consent.The selected treatment is subsequently executed in accordance with evidence-based clinical protocols and standards. For example, while choosing a dental filling, the dentist uses materials and processes substantiated by empirical study.

The researchers developed multiple scenarios for clinicians and students based on the EBD. Each scenario comprises search activities and decision-making tasks. The participants were asked to answer three out of five clinical scenarios. The clinical scenarios were cases from different specialties that might cause some dilemmas for the general practitioner in the clinic. The clinical scenarios included the following specialities: Oral Surgery, Periodontics, Endodontics/Pedodontics, Operative, and Orthodontics. For each scenario answered, the participant was asked to justify the reason for the chosen answer according to the options. The options were ***(1)*** personal experience, ***(2)*** reading it from a book, ***(3)*** reading it online, ***(4)*** discussing it with a colleague, and ***(5)*** other. In addition, they were informed to access the Internet, search for answers for the cases presented, and provide evidence for their chosen answer. Sites searched, the time needed for searching, and the final solution after internet usage were recorded.

The participants were presented with the same five scenarios a second time, and they were tasked with using the information retrieval system to identify and document the evidence to support their responses to the scenarios in order to complete the exercise. They wanted the participants to think through the scenarios as they would in a clinical setting, thus they did not want them to spend too much time on any one question. Clinicians were informed to answer each question in no more than 10 min. The authors did not track how long it took to do searches in each case but instead used data collected from server logs. Participating clinicians reported when they completed their 80 min. Throughout the session, a researcher was present to provide modest technical support.

In the second part, the participants were asked to attend a 15-min lecture about search strategies and tips to help improve their search skills. It was presented through a Microsoft PowerPoint Presentation. After the lecture completion, the authors conducted the post-test to test the participants' searching skills. During the post-test, they were given the same test form and asked to access the Internet to answer it. They were also required to mention **(1)** how valuable the lecture was to their mode of search and **(2)** if they recommend similar extracurricular lecture(s) during the academic year.

### 2.3 Statistical analysis

The statistical testing was conducted in SPSS version 16 in order to identify the particants’ characteristics. The researchers shared the questionnaire with an IT expert and senior dentist to validate the content. Based on their feedback, they modified the content. Clinicians' pre- and post-Internet written replies to scenario questions were examined. The right response was recorded as "Right" for scenarios answered before accessing the online information system during the pre-test. on the other hand, in the post-test, the correct answer and a relevant proof source were coded as "correct" for scenarios answered after accessing the online information system. In Stage 2, incorrect responses were deemed to have no supporting evidence. Based on the study [4], it was determined how people responded to hypothetical situations before and after they had access to the Internet by classifying their responses according to the following categories:

• Wrong Right (RW): Wrong answer before but correct answer after the lecture.
• Wrong Wrong (WW): Wrong answer before online information retrieval system use and wrong answer after the lecture.
• Right Wrong (RW): Right answer before but wrong after the lecture.
• Right Right (RR): Right answer before and right after the lecture.

The data analysis performed by the students was compared to that performed by the clinicians. We tested their ability to search before and after practicing it. The percentage of correct responses on the initial assessment was compared to that on the final evaluation. The significance of the direction of the change was evaluated using the Sign test. Changes in test scores between the pre and post-test were analyzed using correlation proportion tests for each occupational group and the entire sample.

For this reason, the authors used the McNemar test to examine the trend of shifts between the pre-and post-test results. Characteristics of each professional group's history were compared using analysis of variance. The real duration of each search was extracted from the system logs of the online database retrieval system.

## 3 Results

The researcher invited a total of 98 clinicians (60 interns, 15 demonstrators, and 23 postgraduates [AEGD, Board, and Specialists]). 58 clinicians were invited from the Dental College of King Saud University (KSU. In addition, 25 clinicians were contacted from Riyadh Kharj Hospital (RKH), and 15 from Riyadh Medical Center (RMC). Of the total 98 clinicians, 52 were females, and 46 were males. From the 98 clinicians, only 37 answered the queries. Table 2, Table 3 reveal the response rate was 37.8 %, showing the details for the different groups of clinicians.

**Table 2:** Response rate according to different groups.

Type of Clinicians	Invited Clinicians	Tests Answered	Response Rate %
Interns	60	34	56.6
Demonstrators	15	3	20.0
Others^a^	23	0	0
Total	98	37	37.75

a Others: A.E.G.D., Board, Specialists.

**Table 3:** Response rate according to gender.

Gender	Invited Clinicians	Tests Answered	Response Rate %
Female	52	19	36.53
Male	46	18	39.13
Total	98	37	37.75

The number of students contacted was 70 (35 third-year students, 15 fourth-year students, 20 fifth-year students). Of the 70 students who took the test forms, only 23 were returned; the response rate was 32.8 %. Table 4 shows the response rate for the different academic levels.

**Table 4:** Response rate according to academic year.

Year of course	Tests Distributed	Tests Answered	Response Rate %
3rd year	35	9	25.71
4th year	15	6	40.0
5th year	20	8	40.0
Total	70	23	32.85

At the beginning of the study, 71 % of the 60 clinicians and students rated their computer skills as good to excellent, and 46.7 % reported using an online information retrieval system once weekly or more frequently. The clinicians and students provided 180 clinical scenario answers (111 from clinicians and 69 from students).

### 3.1 Searching strategies

For pre-internet use, clinicians and students correctly answered 58.3 % of scenario questions. Table 5 outlines the changes in the direction of the answers. For 55 answers, a correct response was recorded pre-internet usage; however, post-internet use, no evidence was recorded to support their response. An incorrect pre-test answer was recorded in 52 scenario answers, and no post-test response was given.

**Table 5:** Changes in scenario answers pre- and post-internet use.

Scenario Responses			
Pre-internet use	Post-internet use	Total Number	Percentage
Wrong	Wrong	52	28.9
Wrong	Right	23	12.8
Right	Wrong	55	30.5
Right	Right	50	27.8
Total		180	100 %

### 3.2 EBD and IRS skills

Table 6 states that only 40.5 % were able to document a relevant evidence source. Students' documented searching time ranging from a couple of minutes to 30 min, while clinicians' reached up to an hour and a half. Table 7 outlines the mean time that students took to search using the information retrieval system per scenario was 9 min, whereas clinicians took 16 min.

**Table 6:** The percentage of evidence provided.

	Number of Answers	Percentage %
Relevant Evidence	73	40.5
No or Irrelevant Evidence	107	59.5
Total	180	100 %

**Table 7:** Mean time needed to answer each clinical scenario for clinicians and students.

	Clinicians time/Mins	Students time/Mins
1st Scenario	17.1	10.4
2nd Scenario	13.5	7.6
3rd Scenario	17.7	9.7
Total Mean	16.1	9.2

The authors requested the students to participate in the pre- and post-test, and subsequent lectures on search strategies. However, 23 students responded to the pre-test, whereas 10 were involved in the post-test and attended the lecture. The post-test participants found the lecture helpful and recommended similar lectures in college (extracurricular). After a data analysis of the sample of 10 students, we found that the percentage of evidence provided in the "pre-test" was 60 %. This percentage improved in the post–test to 73.3 % (Table 8). The mean time used to search for the answers to the three clinical scenarios in the "pre-test" was almost 10 min, and this meantime improved in the "post-test" to be 8.9 min (Table 9). This low response rate of 43.5 % can be due to the limited time available, the common interest in attending lectures and learning new things, and continuous examinations and clinical requirements.

**Table 8:** Percentage of evidence provided in pre- and post tests.

	Pre-Test	Post-Test
Relevant Evidence	18 (60 %)	22 (73.3 %)
No or Irrelevant Evidence	12 (40 %)	8 (26.7 %)
Total	30 (100 %)	30 (100 %)

**Table 9:** Mean time needed to answer each clinical scenario in pre- and post tests.

	Pre-time/Minutes	Post-time/Minutes
1st Scenario	11.8	11.3
2nd Scenario	7.6	9
3rd Scenario	11.3	6.5
Total Mean	10.2	8.9

## 4 Discussion

In this study, the authors evaluated the dental clinician's and students' knowledge of online information retrieval systems. 98 clinicians and 70 students were invited to participate in the research. Pre- and post-test were conducted in order to identify any significant improvements in the participant's knowledge of information retrieval. The study outcome is similar to the study's findings [[2], [3], [4]], which investigated information retrieval and awareness of evidence-based dentistry. Several studies [[5], [6], [7], [8], [9]] have reported on information technology and the use of the Internet in dental education and their potential use as an educational tool. This study showed clinicians and dental students have a low baseline knowledge of the Online IRS.

In recent years, there has been a shift in dental care toward placing a premium on drawing from the highest quality research to customize treatment plans to the unique needs of each patient. The current study aimed to determine how dental clinicians and students use information retrieval systems to learn new scientific material and how well they understand how to appraise relevant studies while answering clinical issues critically. Many students relied on one another, the Saudi digital library for dental practitioners, and their lecture notes as their primary sources of information. Even though they rarely read scientific publications, students generally remained confident in their ability to retrieve relevant information from the literature. Clinicians also rely on electronic resources like Pubmed, Medline, etc. Students' understanding of the online information retrieval system was insufficient for them to critically analyze clinical research findings, as shown by their responses to three questions about the evaluation of evidence in dentistry.

Students learn from both their peers and their teachers. Our research lends credence to the idea that peer interaction is crucial to the education of adult students, which should be taken into account while designing fundamental dentistry programs. When researching dental-related topics for class, students frequently turned to the Saudi digital library site. All dentistry students in Saudi Arabia have 24/7 access to this site, both on campus and at home. Students and educators in Saudi Arabia make extensive use of the Portal, the country's digital library. For instance, the King Saud University healthcare site receives over 5000 daily visits from students enrolled in the university's medical and dentistry schools. Students accessed evidence-based medical resources (such as the Cochrane Library, MEDLINE, and scientific publications) less frequently. This is significant because the most recent findings published in the scientific literature should serve as the primary source for tasks requiring students to do literature reviews, solve problems, conduct information searches, or critically evaluate treatment procedures.

Based on our findings, students who study at home are more likely to use resources like MEDLINE and dentistry journals. Students can enter the workforce after completing a 5-year dentistry program in Saudi Arabia. They typically want guidance from more experienced peers. In an emergency setting, it is not practical to undertake a comprehensive literature review and meta-analysis to find a solution to a patient's condition. Therefore, more experienced colleagues, the Saudi digital library's in-depth review articles, and the Current Care Guidelines are all excellent resources for rapidly gaining access to critical knowledge in emergencies.

Table 1, Table 2 present the number of clinicians and students involved in the study. It is evident that the number of samples is lesser in this study. It is common practice in pre- and post-test designs for the same group of individuals to take the tests before and after the intervention. The utilization of a within-subject design has the potential to yield greater statistical efficiency compared to a between-subjects design, whereby distinct groups are assigned varying treatments. Utilizing a within-subject design entails employing the same participants as their controls, mitigating the necessity for a substantial sample size. The conduct of research, most notably in clinical or educational settings, may be hindered by restricted resources, such as time, finance, and access to participants. This can be especially problematic in educational settings. Thus, the Researchers are able to use reduced sample sizes as a result of practical constraints.Pre- and post-test designs focus on alterations or disparities within subjects within a specific period. These designs demonstrate enhanced efficacy when researchers anticipate substantial and consistent benefits within the same group of participants. Consequently, smaller sample sizes may still possess statistical power to identify significant changes.

Of the 98 clinicians, only 37 returned it with answers (the response rate was 37.8 %). The low response rate was possibly due to the length of the form and the time consumption, making it difficult for the clinicians to find the time to answer it. In addition to those reasons, the students had several weeks of examinations, clinical requirements, and then the mid-year vacation, which led to a low response rate of 32.8 %. A similar study stated that the main obstacles identified by students as barriers to using the Internet were time and availability of computers.In the test form, 71 % of the 60 clinicians and students rated their computer skills as good or excellent, and 46.7 % reported using an online information retrieval system once or more frequently per week. Neither the reported frequency of online information retrieval nor computer skills were associated with better performance in the experiment (as measured by the number of correct post-internet use answers).

After the examination of the changes in the direction of answers, we noticed that 28.9 % of clinicians and students had no change in their incorrect answers, and 30.5 % changed their answers to be incorrect, possibly due to their poor knowledge in searching for information using the Internet and their inability to find a source of evidence. We also noticed that 12.8 % of clinicians and students could correct their answers and provide proof of their updated answers. In comparison, 27.8 % confirmed they were correct answers after internet usage and provided evidence as well. Only 40.5 clinicians and students could find sufficient evidence to answer their clinical questions or support the chosen solutions. This ability can be due to previous knowledge and training in internet searching. The most significant factors associated with successful question answering were being a medical student, knowing the answer ahead of time, having a higher standardized test score, and having more literature-searching experience. After analyzing the data and calculating the mean time used by clinicians and students to search the Internet, we found that clinicians recorded 16.1 min, a longer mean time than students, who recorded 9.2 min. This might be due to the wide range of the time used by clinicians, as their time ranged from a few minutes to about an hour and a half, but students used similar time records, which makes this difference insignificant.

The post-test outcome reveals that the students found the lecture helpful and recommended similar studies to be given in college (extracurricular). The authors identify that an intervention of introducing students to helpful Websites and practicing answering clinical questions could reduce the effort needed to find answers while improving the quality of the responses found. The percentage of evidence provided in the "pre-test" was 60 %, which improved in the "post–test" to 73.3 %. The mean time used for internet search in the "pre-test" was almost 10 min, which improved in the "post-test" to 8.9 min. This improvement in evidence finding and time of search proves the effectiveness of the lecture and the continuous need for similar courses that improve the student's skills and knowledge of internet information retrieval systems.

Based on our findings, it appears that dental students did not place a high priority on identifying and reading scientific articles, a critical skill for evidence-based dentistry. The outcome follows the study findings [[10], [11], [12], [13], [14], [15]]. Moreover, similar to the findings of [[16], [17], [18], [19], [20], [21], [22], [23]], this study believes that information retrieval systems can support students and clinicians to focus on evidence-based dentistry. These research results agree with those of related medical disciplines [[24], [25], [26], [27], [28]]. There are several reasons why students might struggle to read scientific papers, including a lack of familiarity with the terminology and research methods used in these papers, a lack of understanding of statistics, and a lack of training to recognize the clinical application of knowledge gained from scientific journal articles. Previous research has also indicated that dentistry students lack access to resources for collecting evidence-based knowledge hinders progress in this area. They were optimistic, though, and promised to use it.

Saudi Arabian students scored higher because they were more invested in their research projects, which required them to draw heavily from the literature. Students in their final year performed better than those in their previous year because they could finish their study and write a comprehensive report using a literature search to back up their claims. Even while dentistry students have been praised for their meta-analysis skills, it is troubling that they lack knowledge of searching tactics. Students may have forgotten these abilities since they were assigned early in the program, in the first and second preclinical years. Therefore, it is crucial to stress the significance of search tactics across the whole curriculum. However, evaluating students' approaches to learning was not the primary goal of our study.

According to KSA assessments, students' recall abilities are generally strong. As they progress through their coursework, students develop a solid disposition for obtaining material independently through databases, likely the underlying cause. In their replies, students may have prioritized information retrieval in order to solve clinical and practical problems above exploring scientific evidence. Like their counterparts, Saudi dentistry students should be involved in conducting original scientific research as part of their curriculum.

The curriculum is subject-based, while many schools have transitioned to or are working towards a more integrated approach. Students are exposed to research during the preclinical years when they complete a mentor-guided project. The project enables them to retrieve and analyze information. In addition, they should conduct research before attending seminars and case-based learning sessions.

## 5 Conclusion

This study investigated the role of online information systems in the field of dental sciences. A total of 37 clinicians and 23 students participated in the study. The experimental outcome reveals that 40.5 % of participants were able to extract the evidence while the remaining participants could not produce valid evidence for the search results during the pre-test. On the other hand, in the post-test, there is a significant improvement in the participant's expertise in producing valid evidence. Students demand an extracurricular activity on search strategies to improve their extraction process. The clinicians state that they require a machine learning-based system for understanding the search queries and retrieving relevant evidence to support the decision-making process. The usage of an online information retrieval system was related to a considerable improvement in the quality of responses supplied by clinicians in typical clinical contexts. This improvement can be attributed to the fact that more information is available to clinicians. Moreover, the study's outcome can support the educational organization and healthcare centers to enrich the infrastructure and curriculum to provide an effective working and learning environment.

## Declaration

The authors thank AlMaarefa University for providing the IRB approval (IRB23-019) for conducting this research.

## Data availability statement

Data will be made available on request.

## CRediT authorship contribution statement

**Samar A. Alsaleh:** Writing – review & editing, Writing – original draft, Validation, Methodology, Formal analysis, Data curation, Conceptualization. **Abeer S. Alzawawi:** Writing – review & editing, Writing – original draft, Visualization, Validation, Supervision, Conceptualization. **Alanood A. Alzuhair:** Supervision, Software, Resources, Conceptualization. **Sara A. Kalagi:** Project administration, Methodology, Formal analysis, Data curation, Conceptualization. **Ebtissam M. Al-Madi:** Software, Methodology, Investigation, Funding acquisition, Formal analysis, Data curation, Conceptualization. **Ashit Kumar Dutta:** Software, Resources, Project administration, Methodology, Investigation, Formal analysis, Data curation, Conceptualization.

## Declaration of competing interest

The authors declare that they have no known competing financial interests or personal relationships that could have appeared to influence the work reported in this paper.
